# Changes over time in early complementary feeding of breastfed infants on the island of Hispaniola

**DOI:** 10.26633/RPSP.2017.39

**Published:** 2017-03-22

**Authors:** John D. McLennan

**Affiliations:** 1 Children’s Hospital of Eastern Ontario Research Institute and University of Ottawa Children’s Hospital of Eastern Ontario Research Institute and University of Ottawa OttawaOntario Canada Children’s Hospital of Eastern Ontario Research Institute and University of Ottawa, Ottawa, Ontario, Canada.

**Keywords:** Breast feeding, infant nutrition, milk, human, weaning, Haiti, Dominican Republic, West Indies, Lactancia materna, nutrición del lactante, leche humana, destete, Haiti, República Dominicana, Indias Occidentales, Aleitamento materno, nutrição do lactente, leite humano, desmame, Haiti, República Dominicana, Índias Ocidentais

## Abstract

**Objective.:**

To describe and contrast early complementary feeding (ECF) over time in breastfed infants in the Dominican Republic (DR) and Haiti, the two countries that share the island of Hispaniola.

**Methods.:**

Secondary data analysis was conducted on cross-sectional data from Demographic and Health Surveys administered at four different time-points in both countries between 1994 and 2013. Extracted samples were composed of breastfed infants < 6 months of age whose caregivers had responded to dietary questions on food consumption in the previous 24 hours.

**Results.:**

Plain water was the most frequently consumed complementary substance in both countries. However, the prevalence of water consumption increased in the DR over time, whereas in Haiti it decreased. Milk (non-breast) use was also common and followed a similar pattern as water over time in the two countries. Expanded use of water and milk in the DR are the major contributors to its drop in exclusive breastfeeding (EBF) rates over time. Whereas in Haiti, a reduction in a broader array of liquids and semi-solids/solids overtime appears to have contributed to its markedly improved EBF rates.

**Conclusion.:**

Determining contributors to the differential trends in water and milk (non-breast) use between these two countries may identify targets for addressing the persistent gaps in EBF on the island of Hispaniola.

## RESUMEN

**Objetivo.:**

Describir y contrastar la alimentación complementaria temprana a lo largo del tiempo del lactante amamantado en la República Dominicana y Haití, los dos países que comparten la isla La Española.

**Métodos.:**

Se realizó un análisis secundario de datos transversales obtenidos de las encuestas de demografía y salud administradas en cuatro momentos diferentes en ambos países entre el año 1994 y el 2013. Las muestras extraídas estaban constituidas por lactantes amamantados < 6 meses de edad respecto a los cuales las personas encargadas de su cuidado habían respondido a preguntas sobre el consumo de alimentos en las 24 horas precedentes.

**Resultados.:**

El agua fue la sustancia complementaria consumida con mayor frecuencia en ambos países. Sin embargo, con el transcurso del tiempo fue aumentando la prevalencia del consumo de agua en la República Dominicana, en tanto que disminuyó en Haití. También era común el consumo de leche (no materna), con una tendencia similar a la del consumo del agua en los dos países. El mayor consumo de agua y leche (no materna) en la República Dominicana es uno de los principales factores de la disminución de las tasas de lactancia materna exclusiva con el transcurso del tiempo en ese país. En Haití, en cambio, la reducción de una variedad más amplia de líquidos y semisólidos/sólidos a lo largo del tiempo parece haber contribuido a tasas claramente más altas de lactancia materna exclusiva.

**Conclusiones.:**

Los factores determinantes que contribuyen a las tendencias diferenciales en el consumo de agua y leche (no materna) en estos dos países pueden servir para establecer metas a fin de abordar las brechas que persisten en cuanto a la lactancia materna exclusiva en la isla La Española.

## RESUMO

**Objetivo.:**

Descrever e examinar a evolução temporal da introdução precoce da alimentação complementar em lactentes amamentados na Ilha de São Domingos (formada pela República Dominicana e Haiti).

**Métodos.:**

Uma análise de dados secundários foi conduzida com dados transversais coletados em pesquisas de demografia e saúde realizadas República Dominicana e Haiti em quatro pontos temporais distintos entre 1994 e 2013. As amostras obtidas incluíram lactentes amamentados menores de 6 meses de idade cujos cuidadores responderam um questionário sobre consumo alimentar nas 24 horas precedentes.

**Resultados.:**

Água pura foi a substância complementar mais consumida em ambos os países. Porém, a prevalência do consumo de água aumentou com o tempo na República Dominicana, enquanto diminuiu no Haiti. Foi observado também o consumo de leite (não materno) que seguiu um padrão temporal semelhante ao da água nos dois países. O amplo consumo de água e leite (não materno) na República Dominicana é um dos principais contribuintes para a queda do índice de aleitamento materno exclusivo (AME) ao longo do tempo neste país. No entanto, no Haiti, a redução no consumo de uma ampla variedade de alimentos líquidos e semissólidos/sólidos com o tempo parece ter contribuído para uma melhora acentuada no índice de AME.

**Conclusão.:**

Determinar os contribuintes para as tendências diferenciais de consumo de água e leite (não materno) entre os dois países pode ajudar a definir as metas visando sanar as lacunas persistentes em AME na Ilha de São Domingos.

The recommendation for exclusive breastfeeding (EBF) for the first months of life has broad support given its relationship with lower child morbidity and mortality (1, 2). Although debate continues as to whether the World Health Organization’s 6-month recommendation for EBF (3) should be applied to all countries, there is strong support for its application to resource-limited areas with high rates of infections, the case of many low- and middle-income countries (LMIC) (4, 5). Despite this, it is estimated that only 47% – 57% of infants in LMICs are exclusively breastfed for even 2 months (6).

Whereas breastfeeding patterns have been extensively studied across countries and over time (7), early complementary feeding (ECF), i.e., before 6 months of age, has received less scrutiny despite its role in undermining EBF. Although a measure of any ECF can be derived from EBF rates, such an indicator provides little detail on the complex patterns of complementary feeding. For example, a study of Cambodia reported a marked increase in EBF in the first 6 months between surveys in 2000 (11.1%) and 2010 (73.5%); however, changes in complementary feeding contributing to this marked change were not reported (8).

There are exceptions to this information deficit in LMICs. Mexican studies describe changes in EBF prevalence for children < 6 months of age at three time points, 20.0% (1996), 22.3% (2006), and 14.4% (2012), with the recent drop attributed to increased formula and water consumption (9, 10). A study in Rio de Janeiro, Brazil, in 1998 – 2008, found significant reductions in the use of four food types (water/tea; fruit/fruit juices, milk [non-breast], any other food) for children < 6 months (11).

Similarly, this study of the island of Hispaniola, shared by the Dominican Republic (DR) and Haiti, examines ECF practices that may undermine EBF over time. It is proposed that contrasting patterns in the DR and Haiti may be of interest. Surprisingly, there are few investigations contrasting health practices in these two countries, which not only share the same island, but have a complicated, intertwined history (12, 13). Although the DR and Haiti also share many other features—weather, fauna and flora, and population size (both around 10 million)—they also differ. Beyond language differences, per capita gross domestic product (GDP) may be their most striking difference. In 2013, per capita GDP for the DR was estimated at current US$ 6 027 (upper-middle income classification), in contrast to Haiti at US$ 810 (low income classification) (14).

These countries also differ on at least some infant feeding measures. One report noted that the DR had one of the lowest EBF rates among infants 0 – 3 months of age compared with a selection of other Latin American countries (15). A downward trend in EBF in the DR over time, from 25% in 1996 to 12% in 2007, was also noted (15). This contrasted with an increase from 32% to 49% in Haiti between 2000 and 2005/2006 for infants 0 – 3 months of age (15). Examination of ECF in these two countries over time may provide an understanding of complementary feeding practices that underlie these variations in EBF over time on Hispaniola.

## MATERIALS AND METHODS

This was a secondary data analysis study of cross-sectional surveys that are conducted periodically in the DR and Haiti.

### Study samples

In both countries, Demographic and Health Surveys (DHS) are conducted using a two-stage sampling process—representative samples of households within representative samples of enumeration areas (16, 17). Within selected households, women 15 – 49 years of age who have young children are invited to complete an interview that contains a series of questions on infant feeding practices.

The study’s samples were drawn from the four most recent cross-sectional DHS studies in the DR (1996, 2002, 2007, 2013) and in Haiti (1994/1995, 2000, 2005/2006, 2012). Extracted samples were composed of children who were: (i) living in selected households and were < 6 months of age, *and* (ii) alive at the time of the survey, *and* (iii) still being breastfed at the time of the survey, *and* (iv) whose mother had responded to survey questions on food consumption. The sample was restricted to those currently breastfeeding since the study aim was to examine complementary feeding practices that undermine EBF. Final samples sizes for each dataset are summarized in Table 1.

### Ethics

This study was based on publically available datasets that have no personal identifying information. DHS studies receive research ethics approval within host countries.

### Measures

Datasets covered four survey time periods corresponding with DHS phase 3 – phase 6. Although there is some consistency in the structure and content of feeding questions across surveys, there are specific variations across time and place that are detailed below. Respondents were always asked a “yes/no” question about current breastfeeding. For other dietary practices, respondents were asked about the infant’s food and liquid consumption “yesterday during the day and night” (a 24-hour period) through a series of questions on different food items. The response option to these was “yes/no.” Phase 4 was an exception. It asked the number of times each item was consumed during the 24-hour time period. To facilitate comparison across surveys, the study considered all values > 0 for phase 4 as a “yes” response.

The following were liquids covered by the surveys, and modifications used in this study to facilitate cross-survey comparisons:

**TABLE 1.** Summary of samples sizes with application of inclusion criteria across datasets for a study of complementary feeding of breastfed infants in the Dominican Republic (DR) and Haiti, 1994 - 2013

Application of inclusion criteria^a^	DR 1996 n	DR 2002 n	DR 2007 n	DR 2013 n	Haiti 1994 – 1995 n	Haiti 2000 n	Haiti 2005 – 2006 n	Haiti 2012 n
Child < 6 months of age	433	1 046	1 029	320	338	585	627	767
Child alive at time of study	422	1 024	1 002	313	319	570	603	737
Currently breastfed^b^	348	835	784	234	307	565	591	711
Final sample (unweighted)^c^	332	824	703	233	298	549	587	700
Final sample characteristics:								
Child age in months: mean	2.4	2.4	2.6	2.5	2.6	2.7	2.7	2.9
(standard deviation)	(1.6)	(1.5)	(1.6)	(1.6)	(1.6)	(1.6)	(1.6)	(1.6)
Female (%)	45.5	50.0	45.0	53.6	49.0	47.4	49.1	49.7
Weighted sample size (*n*)	307	751	608	201	307	516	553	662

a Each selection factor applied to resulting subset going from top to bottom.

b Weighted % currently breastfed among live children < 6 months of age: 81.9% (DR-1996); 78.7% (DR-2002); 76.1% (DR-2007); 69.2% (DR-2013); 96.4% (Haiti 1994/5); 98.9% (Haiti 2000); 97.8 (Haiti 2005/6); 95.8% (Haiti 2013).

c Caregiver responded to all questions about infant’s food and liquid consumption in previous 24 hours.

***Source:*** Prepared by the author based on analysis of Demographic and Health Survey data from 1994 - 2013.

- No changes in the original coding were made for the following: *plain water, carbonated water drinks, infant formula, juice, rice water, sugar water, soup/clear broth, and “other liquids.”* While “other liquids” was not modified, cross-time and country comparisons are limited given that the number of specific liquids considered in each survey varied. This is partly addressed by an “any liquid” variable described below.
- Some changes were made for the following:
  *Tin, powder, and/or fresh (TPF) milk:* These were covered in all surveys, however, in Phase 3, two questions were asked (i.e., “tin/powder” and “fresh”), which were combined in this study to facilitate comparison.
  *Tea +/-coffee:* A combined category was created given the variations in questions on these two beverages.
- The following variables were newly created:
  *Combined milk:* Combined “infant formula” and “TPF milk”
  Any liquid other than plain water and milk
  Any liquid including plain water and milk

The following summarizes semi-solid/solid type foods and their modifications:

- No changes in the original coding for the following*: foods made from grains; foods made from legumes; tubers/roots; orange/yellow vegetables; dark green leafy vegetables; vitamin A rich fruits; foods made with oil, fat, butter; “baby cereal”, “other fruits (+/-vegetables).”* The same issues for “other fruits (+ - vegetables)” applies as for “other liquids” noted above.
- Some changes were made in the following:
  *Animal Source* (non-dairy): In some surveys, various source foods were asked in separate stand-alone questions (e.g., poultry, eggs), whereas other surveys combined these into a single question. To facilitate comparisons, all non-dairy animal source foods were collapsed into a single variable.
  *Porridge:* Different questions on porridge and baby cereal were asked inconsistently over time. In phase 3, the Haiti questionnaire used the Kreyol term “labouyi,” which can be translated “porridge;” however, an equivalent question was not included in this phase for the DR. Neither country had a stand-alone porridge/baby cereal question in phase 4. Two different questions were used in phase 5: one specific to commercial baby cereal, and a second that captured any other porridges. In phase 6, only the commercial baby cereal question was asked. To allow comparison of results from the consistent commercial baby cereal question across phases 5 and 6, this variable was not collapsed.
  *Other dairy* (non-milk): Most surveys included a question about cheese, yogurt, and other dairy products (other than milk). The most recent Haiti survey had a stand-alone question on yogurt that was collapsed into this new “other dairy” variable.
  *Other semi-solids/solids:* A specific “other semi-solids/solids” questions was asked after specific category questions in five of the surveys; however, given the more substantial variation in specific semi-solid/solid questions across surveys, this category was modified to include the original “other semi-solid/solid,” as well as the individual semi-solid/solid questions that were only asked in a single survey, i.e., *compote* and *mashed fruit* (DR 1996), and *solid food for child* and *food from family plate* (Haiti 1994/1995).
- The following variables were newly created:
  *Any semi-solid/solid:* This counted any response to semi-solid/solid questions in a given survey.
  *Any liquid or semi-solid/solid:* This collapsed “any liquid including plain water and milk” AND “any semisolid/solid.”

In addition, data were coded to generate levels of breastfeeding (2):

- *Exclusively breastfed:* Nothing other than breastmilk (except for medicine, vitamins, minerals, and Oral Rehydration Solution); operationalized in this study as a “yes” response to current breastfeeding question *and* “no” response to all questions about foods or liquid given in the last 24 hours.
- *Predominately breastfed:* breastmilk *and* (water *and/or* water-based drinks [excluding food-based liquids]); operationalized in this study as a “yes” response to current breastfeeding question *and* a “yes” response to any of the liquid questions except milk *and* a “no” response to milk and all semi-solid/solid food questions in the last 24 hours.
- *Partially breastfed:* breastmilk *and* any semi-solid/solid foods or food-based liquids; operationalized in this study as a “yes” response to current breastfeeding questions and a “yes” response to any milk and/or semi-solid/solid food item.
- *Not breastfed*: Although this group is not included in the analysis, the percentage of children not breastfed at the time of the survey can be derived from values provided in a footnote to Table 1.

### Analysis

Prevalence values for the above variables were generated using DHS weighting to account for sampling design and to allow population level estimates.

## RESULTS

### Early liquids in the DR

Plain water is the most common liquid given to breastfed infants in the DR (Table 2). Across all surveys, the majority of infants also received milk (non-breast), with a steady increase over time, reaching 79.3% in the most recent survey. If the apparent spike in use of both milk types in 2007 is assumed to be an error, then there may be some trend for infant formula to be replaced by non-infant formula milks over time. The collapsed variable “any liquids” identifies an already high use in 1996 that has climbed over time.

### Early liquids in Haiti

While plain water is also the most frequent liquid consumed by breastfed infants in Haiti, there is a marked drop over time, more specifically -41.9% from the 1994/1995 survey to the 2005/2006 survey, with a slight increase in the most recent survey (Table 2). Combined milk (non-breast) has shown a steady decline across all surveys, although no discernable pattern is noted between changes in infant versus non-infant formula milk over time. Other specific liquids considered on at least two occasions generally demonstrate a decreased use over time as well. Sugar water use was particularly high (29%), the one time it was asked (1994/1995). Theoretically, sugar water should be captured by responses to the “other liquid” question in the other surveys. The collapsed “any liquid” categories also capture the generally consistent downward trend of complementary liquid use over time in Haiti, which contrasts sharply with the DR.

**TABLE 2.** Frequency of use of complementary liquids in the Dominican Republic (DR) and Haiti, 1994 - 2013^a^

Liquid type	DR 1996 %	DR 2002 %	DR 2007 %	DR 2013 %	Haiti 1994/1995 %	Haiti 2000 %	Haiti 2005/2006 %	Haiti 2012 %
Plain water	55.4	66.4	73.6	71.4	95.5	72.1	53.6	56.7
Infant formula	41.6	43.9	69.2	33.6	18.1	21.9	13.8	16.6
TPF milk^b^	13.7	25.7	70.0	51.2	22.0	8.7	20.1	3.7
Combined milk^c^	54.7	69.4	73.4	79.3	35.7	30.1	27.8	19.6
Juice	23.7	12.0	NA	19.8	19.2	15.1	7.1	9.2
Tea (+/-coffee)	25.9	NA^d^	17.0	NA	22.6	NA	8.4	NA
Rice water	7.3	NA	NA	NA	NA	NA	NA	NA
Soup/clear broth	6.7	NA	NA	3.2	NA	NA	8.8	3.9
Sugar water	4.3	NA	NA	NA	29.0	NA	NA	NA
Carbonated water drink	NA	NA	NA	NA	0.7	NA	NA	NA
“Other liquids”	5.5	12.9	9.3	12.8	3.3	14.7	4.6	7.6
Any liquid other than plain water and milk	46.6	21.1	23.2	32.0	47.1	25.8	20.7	18.0
Any liquid including plain water and milk	76.6	86.5	88.6	90.5	96.9	75.3	58.1	57.6

a Percentages based on weighted sample.

b Tin, powder, and/or fresh milk.

c Combined milk is the combination of TPF and infant formula.

d Not asked in the given survey.

***Source.*** Prepared by the author based on analysis of Demographic and Health Survey data from 1994 - 2013.

### Early semi-solids/solids in the DR

There are less distinct trends for the introduction of semi-solid/solid use over time (Table 3). Comparing only the first and last surveys, use of foods made from grains and tubers decreased. Legume and orange/yellow vegetables appear somewhat stable over the three times they were considered. Most other specific food categories were investigated infrequently or were consumed infrequently (< 5.0%). Baby cereal was used for 8.3% of infants when it was first asked as an explicit question (2007), although it has since dropped in prevalence. “Compote” was the major contributor to the revised “other semi-solid/solid” variable in the first survey. Compote was not subsequently asked as a stand-alone question, but theoretically should be captured within the “other semi-solid/solid” question asked in the other surveys. There is no clear trend in the “any semi-solid/solid” variable over time, however, comparability may be limited given variation in items considered across surveys.

### Early semi-solids/solids in Haiti

Unlike the DR, use of baby cereals and porridge appears to be more substantial. On the two surveys that asked this question, the frequency of porridge use was high. There is a data gap created by the fact that not all the surveys consistently asked this question. The drop between the 1994/1995 and 2005/2006 surveys, which both used the term “labouyi,” is substantial (29.1%). A number of categories that were covered in the last three surveys demonstrate a consistent drop in 2005/2006, with subsequent increase in the last survey. In contrast, “other dairy” and legume use have shown some increase over time.

There is a marked drop in “any semisolid/solid” use between the first and second surveys. To what extent this change is a function of change in practice versus a function of question change is not discernable from the data; however, “any semi-solid/solid use” continues to be reported by a substantial minority and is relatively stable over the last two surveys.

### Breastfeeding patterns in the DR

While the majority of infants < 6 months of age were reported as breastfed across all surveys, the rate has been consistently dropping over time with a change of -12.7% from the first to last survey (Table 1, footnote b). Among those breastfeeding, only a minority are classified as exclusively or predominately breastfed, and these values have been consistently dropping over time, with a concomitant increase in the partially breastfed group (Table 4). Plain water and milk are major and unique contributors to low EBF prevalence across time, particularly for those who used both. For example, based on 1996 data, if milk and water are not counted in determining EBF, its prevalence would change from 22.6% to 47.8% (an increase of 25.2%), and in 2013, the change would be from 9.5% to 60.0% (an increase of 50.5%).

### Breastfeeding patterns in Haiti

The vast majority of Haitian infants < 6 months of age have been breastfed over time (> 95% in every survey) (Table 1, footnote b). In contrast to the DR, among those breastfeeding, there has been a marked increase in the EBF subgroup across the first three surveys, with a leveling off in the most recent survey (Table 4). The unique impact of not counting plain water and milk in determining EBF rates within Haitian data has a smaller impact than in the DR, although the contribution was substantial (29.7%) in 2000. Rather than being a function principally of changes in water and milk use alone, the increases in EBF rates in Haiti appear to be a function of drops in a broad array of liquids and solids used at earlier survey time points. However, this interpretation is tempered, given gaps in information on trends on some specific food types (e.g., porridge and sugar water).

## DISCUSSION

Despite the shared characteristics of the two countries, there are a number of differences in infant feeding over time. Haiti consistently has had higher breastfeeding rates than the DR. The most recent surveys demonstrate further separation in both the overall breastfeeding rate and the EBF fraction. Complementary feeding patterns drive the difference in the EBF fraction, with plain water and milk playing a particularly dominant role in the DR, while a reduction in a broader array of complementary liquids and foods in Haiti over time has fueled the expanded EBF pattern in that country.

**TABLE 3.** Frequency of use of complementary semi-solids/solids in the Dominican Republic (DR) and Haiti, 1994 - 2013^a^

Food type	DR 1996 %	DR 2002 %	DR 2007 %	DR 2013 %	Haiti 1994/1995 %	Haiti 2000 %	Haiti 2005 /2006 %	Haiti 2012 %
Grains, foods made from (e.g., bread, noodles)	11.6^b^	3.6	7.0	3.9	NA^c^	16.7	10.4	14.0
Tubers/roots (e.g., potatoes, cassava)	5.0	2.1	3.3	1.4	NA	11.4	1.3	5.3
Legumes, foods made from (e.g., lentils, beans, peanuts, peas)	NA	4.8	7.3	5.8	NA	2.2	2.7	3.1
Orange/yellow vegetables (e.g., pumpkin, carrots)	NA	2.4	4.7	2.7	NA	4.5	1.6	3.7
Oil, fat, butter, foods made with	NA	2.9	4.9	NA	NA	14.3	11.5	NA
“Other fruits (+/-vegetables)”	NA	1.7	1.7	1.3	NA	6.6	4.0	4.8
Animal source (non-dairy)	NA	0.9	4.0	2.2	NA	1.9	2.8	2.5
Vitamin A rich fruits (e.g., mango, papaya)	NA	1.1	3.2	0.2	NA	1.4	0.3	0.9
Dark green leafy vegetables	NA	0.1	0.2	0.0	NA	2.3	0.8	3.7
Other dairy (e.g., cheese, yogurt)	NA	0.1	1.5	0.2	NA	0.6	3.4	6.5
Baby cereal	NA	NA	8.3	4.6	NA	NA	12.6	15.3
Porridge	NA	NA	3.3	NA	54.8	NA	25.7	NA
Other semi-solid/solids	16.1^d^	NA	6.3	5.2	40.0^e^	NA	10.9	22.4
Any semi-solid/solid	21.3	10.2	24.6	17.7	73.8	34.2	40.7	41.7
Any liquid or semi-solid/solid	77.4	87.2	89.9	90.5	97.3	76.0	58.4	58.5

a Percentages based on weighted sample.

b While this value is in the database, the corresponding question was not found in the accompanying Spanish questionnaire; hence, this value should be interpreted with caution as it may be an error.

c Not asked.

d Within this value, 8.8% of the total sample reported consumption of compote or in Spanish “compota” which may be translated “stewed fruit,” although more contemporarily refers to commercial jarred baby food in the DR.

e This captures responses to “solid food for child” and “household food/family plate.”

***Source.*** Prepared by the author based on analysis of Demographic and Health Survey data from 1994 - 2013.

**TABLE 4.** Summary of breastfeeding status in the Dominican Republic (DR) and Haiti, 1994 - 2013^a^

Breastfeeding status	DR 1996 %	DR 2002 %	DR 2007 %	DR 2013 %	Haiti 1994/1995 %	Haiti 2000 %	Haiti 2005/2006 %	Haiti 2012 %
Partially breastfed	60.6	71.4	78.6	81.9	80.6	49.6	46.6	46.3
Predominately breastfed	16.8	15.9	11.4	8.6	16.7	26.3	11.8	12.2
Exclusively breastfed (EBF)	22.6	12.8	10.1	9.5	2.7	24.0	41.6	41.5
EBF if not counting plain water use	28.2	24.5	18.1	15.0	10.5	41.1	50.9	51.0
EBF if not counting milk use	30.6	28.6	21.6	21.3	2.7	25.7	44.0	41.8
EBF if not counting plain water or milk use	47.8	74.4	61.8	60.0	14.2	53.7	56.0	55.2
EBF if not counting any “other liquids” (but counting water, milk, and semi-solids)	28.4	14.3	11.8	11.1	3.1	24.9	42.3	42.0
EBF if not counting any semi-solids/solids	23.4	13.5	11.4	9.5	3.1	24.7	41.9	42.4

a Percentages based on weighted sample

***Source:*** Prepared by the author based on analysis of Demographic and Health Survey data from 1994 - 2013.

What factors influence this ECF pattern divergence is unknown. Both countries have governmental and nongovernmental organizations promoting EBF. However, the quality, population coverage, and impacts of such initiatives are unknown. That the DR was rated 36th of 40 countries—using a measure to rate the extent to which a country has met priority indicators for recommended infant and young child feeding policies and programs—is a concern, especially given that these indicators are related to greater improvement in country level EBF (18, 19). Haiti was not included in that study. Heidkamp and colleagues (20) noted that in Haiti there were specific breastfeeding promotion efforts in the late 1990s that may be related to improved EBF, but also raised the possibility that increased awareness of EBF recommendations could have led to a social desirability bias. It is unknown if levels of awareness of EBF recommendations differ between the DR and Haiti or whether such differences account for actual and/or reported practice differences.

Economic status is a striking difference between the two countries with further divergence over time. To what extent this is a driver of actual feeding differences is unknown, although EBF overall is more common in least developed countries (21). Another difference between these two countries, which may be related to economic differences, is the much higher and dramatically rising caesarean-section (C-section) rate in the DR (25.9% in 1996 and 56.4% in 2013) compared with Haiti (1.6% in 1994/1995 and 5.5% in 2012) (22 – 25). C-sections, particularly pre-labor C-sections, appear to adversely impact at least some aspects of breastfeeding (26).

Despite the differences, plain water consumption is common in both countries. Water has previously been noted as an important factor influencing variation in EBF over time in other Latin American countries (9 – 11, 27). While water use might be rationalized for hydration, at least one study found that supplemental water is not required for breastfed infants, even in hot and humid conditions (28). Further investigation is required to identify caregiver reasoning behind water use in young infants. Of concern, at least in the DR, is the marketing of “special” bottled water for infants. Whether this accounts for some of the increase in early water use over time in the DR is unknown.

Many studies have examined breastmilk versus other milk use. However, it is important to emphasize that the samples considered in this study were all breast feeders, so the measures of prevalence of milk (non-breast) use is within a breastfeeding population. This mixed milk feeding approach has been associated with shorter durations of breastfeeding (29, 30). The higher levels of mixed milk feeding in the DR may be partially a function of higher income levels that may allow for more frequent purchase of relatively expensive food sources, in particular infant formula. However, while milk use is higher in the DR, the relative proportion of infant formula users is actually higher in Haiti (84.7%) than the DR (42.4%) in the most recent surveys.

While water and milk use have been consistently asked throughout the included surveys, other types of liquids (e.g., sugar water) have been more inconsistently asked, making it more difficult to determine patterns. Information on semi-solid/solid use is even less clear. Of particular importance may be baby cereal and porridge use in Haiti. The failure to include a porridge question in the 2012 survey may have resulted in an underestimate of ECF and overestimate of EBF rates. One study included a description of porridge preparations that are fed to infants in Haiti and included a gruel made from salty or sweet crackers cooked with water and sugar; a porridge called *soupe de pain* (bread soup) made with bread, water, salt, and margarine; and *bouillie de farine* (flour gruel) made of plantain, manioc, or white wheat flour (31). How extensively such preparations are used throughout Haiti is unknown. Similarly detailed gruel descriptions were not found for the DR.

### Limitations

There were some study limitations that should be noted. First, while there were a number of consistencies in questions across surveys, there were several exceptions (detailed above). These weaken some of the cross-country and cross-time comparisons. Second, there has been criticism of relying on single cross-sectional 24-hour dietary recall to classify breastfeeding (32), with the potential of overestimating EBF levels (33). Although this will impact absolute rates, it should not directly impact the relative contrasts in this study given the consistent methodological approach over time and across countries. Third, the decision to focus only on feeding practices within breastfed infants does not address the concern of those who are not breastfed. Despite these gaps, sufficient data were available to identify trends over time and differences between the two countries.

### Conclusions

Targeting reduction of early water introduction should be a priority for improving EBF. In this regard, learning how Haiti successfully reduced this practice over time may be informative. Similarly, learning from the drop in early complementary milk use in Haiti may be useful, especially since likelihood of it undermining breastfeeding is greater than water’s. Expansion of data collection within the child nutrition modules of DHS surveys could help discover potential influencing factors. A more tailored nutrition survey module, one specific to infants < 6 months of age, could aid in further exploring and identifying specific patterns, rather than relying on the more generic dietary questions intended for the 0 – 5 year age group. Given the importance of EBF in the early months, such an investment would be reasonable.

### Acknowledgements:

The author wishes to thank the DHS Program for making survey data available for this study and the families from the Dominican Republic and Haiti that participated in the original surveys.

### Disclaimer.

Authors hold sole responsibility for the views expressed in the manuscript, which may not necessarily reflect the opinion or policy of the *RPSP/PAJPH* and/or PAHO.
